# High-Contrast Observation of Unstained Proteins and Viruses by Scanning Electron Microscopy

**DOI:** 10.1371/journal.pone.0046904

**Published:** 2012-10-08

**Authors:** Toshihiko Ogura

**Affiliations:** Biomedical Research Institute, National Institute of Advanced Industrial Science and Technology (AIST), Central 2, Umezono, Tsukuba, Ibaraki, Japan; University of Kansas Medical Center, United States of America

## Abstract

Scanning electron microscopy (SEM) is an important tool for the nanometre-scale analysis of the various samples. Imaging of biological specimens can be difficult for two reasons: (1) Samples must often be left unstained to observe detail of the biological structures; however, lack of staining significantly decreases image contrast. (2) Samples are prone to serious radiation damage from electron beam. Herein we report a novel method for sample preparation involving placement on a new metal-coated insulator film. This method enables obtaining high-contrast images from unstained proteins and viruses by scanning electron microscopy with minimal electron radiation damage. These images are similar to those obtained by transmission electron microscopy. In addition, the method can be easily used to observe specimens of proteins, viruses and other organic samples by using SEM.

## Introduction

To better understand various biological functions, observation of the nanometre structures of proteins and viruses is essential [1]–[3]. Electron microscopy is an important tool for such observations [4]–[6]. Prior to observation, samples are usually prepared by staining to enhance image contrast. However, for proteins and viruses, staining is often not recommended to observe detail of the biological structures. Moreover, biological samples are prone to radiation damage and unstained samples give very poor contrast [6]–[8].

To address these problems, traditional sample preparation techniques such as glutaraldehyde fixation, negative staining and heavy metal coating have been developed [9]–[11] and used for several proteins and viruses [12]. However, the obtained images often include artefacts from staining and/or metal coating. In addition, new unstained imaging techniques based on cryo-stage freezing have been employed for biological specimens [13]–[15]. However, the obtained images have very low contrast and high noise owing to the low radiation dose needed to minimize sample damage [6]. Therefore, the goal of observing unstained proteins and viruses by electron microscopy under high contrast and with low radiation damage remains elusive.

Recently, we reported a new method for preparing unstained bacteria and viruses that involved their placement under thin carbon films, followed by observation with a scanning electron microscope (SEM) [16], [17]. High contrast is accomplished by secondary electrons (SEs) generated in the carbon films, which result in only low radiation damage of unstained biological samples. We call this system the *indirect secondary electron contrast* (ISEC) method [16]. However, the method achieves insufficient resolution for use with proteins, and observation of the inner structures of viruses is difficult.

Herein we report a new method for preparing unstained proteins and viruses by placement under metal-coated insulator films. The obtained images of as-prepared samples show very high contrast, and the samples incur only low electron radiation damage. The mechanism of obtaining images differs from that of the previous ISEC method.

## Results

### SEM System Overview

**Figure 1:**
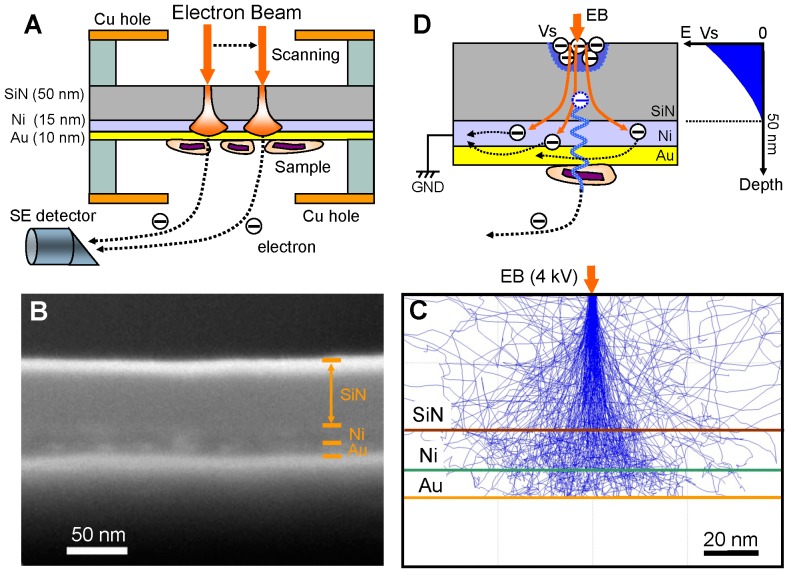
Experimental setup and SEM-based observation method. (A) Schematic diagram of our new SEM-based measurement system. Unstained proteins and viruses are attached to the bottom of the Ni–Au-coated SiN film. A scanning-EB (acceleration 3.6–4.0 kV) irradiates the upper side of the SiN surface. The irradiated electrons scatter and are absorbed into the film. Images of the sample are observed at the bottom of the film. (B) SEM cross-sectional image of the metal-coated SiN film composed of a 50-nm-thick SiN film, 15-nm Ni layer and 10-nm Au layer. (C) Monte Carlo simulation of electron trajectories in the metal-coated film. The EB spot diameter is 3 nm. At an accelerating voltage of 4 kV, almost all electrons that pass through the SiN film scatter and are absorbed into the metal layer. (D) Schematic representation of the high-contrast high-resolution detection mechanism for use with unstained biological samples under a metal-coated SiN film. Because the film is highly insulating and the irradiated position on the SiN film is electrostatically charged, a high electric potential gradient develops in the film, and some secondary electrons are transmitted to the sample by quantum tunnelling caused by the gradient. Scale bars: (B) 50 nm, (C) 20 nm.

Unstained proteins and viruses were deposited onto a metal-coated SiN film (Figure 1A). SiN films are known to be highly insulating (approximately 10^14^ Ω·cm) [18]. The coated film contains three components: a 50-nm-thick SiN film coated by sputter deposition with a 15-nm-thick Ni layer followed by a 10-nm-thick Au layer (Figure 1B). The Ni layer is very flat, which facilitates clear observation of small biological samples. The Au layer prevents oxidization of the Ni layer and is non-toxic to biological samples.

The metal-coated SiN film, positioned with the SiN side up and the Au side down and with the sample mounted onto the down-facing Au side, is irradiated from above with a 4-kV electron beam (EB) from a field-emission SEM (FE-SEM). Monte Carlo (MC) simulations show that numerous irradiated electrons scatter strongly, pass through the SiN film and finally are absorbed by the Ni–Au metal layer (Figure 1C). The sample is not directly irradiated and hence is minimally subject to electron radiation damage.

During irradiation, a few electrons are trapped on the SiN film surface at the EB-irradiated position because the film is highly insulating [18]. However, the Ni–Au layer under the film remains at 0 V because the electrons absorbed into the metal layer are quickly discharged through electrical grounding. Thus, a high electric potential gradient develops in the film (Figure 1D), and this gradient pushes any SEs generated in the film down towards the sample. Then some of these SEs pass into the sample by quantum tunnelling. The SE detector of the SEM measures SE transmission into the sample. Therefore, the observation images presented high-contrast, since SEs that reach the bottom side of the film contain the structural information of the sample.

### Observation of Unstained Baculovirus

**Figure 2:**
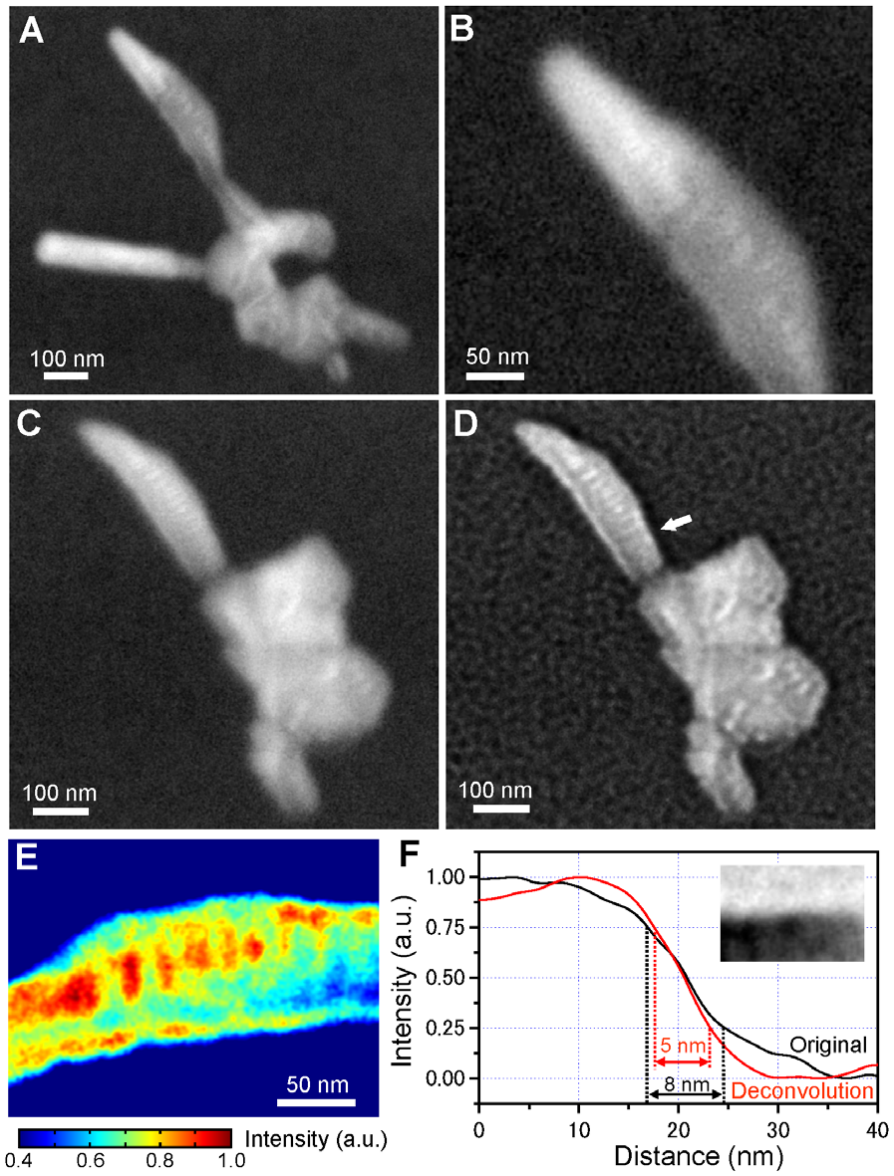
High-contrast images of unstained baculoviure obtained by our new measurement method. (A) Image of unstained baculovirus under a metal-coated SiN film, obtained with an original secondary electron detector of the FE-SEM. The image was taken at 80,000× magnification with a 4-kV EB accelerating voltage, then treated with a 2D Gaussian filter (size 9×9 pixels, σ = 1) after contrast reverse. Two baculoviruses are visible at the top left and the baculovirus envelope is clearly visible at the bottom right. (B) Expanded image of the baculovirus at the top left. A cigar-shaped nucleopside is visible at the centre of the virus body. (C) Image of another unstained baculovirus under a metal-coated SiN film. A similar rod-shaped virion with an envelope is visible. (D) Super-resolution image of (C), obtained by applying the Lucy–Richardson deconvolution algorithm. A virion with an envelope is more clearly visible than in the original image. (E) Expanded pseudo-colour map of the virion centre in (D). Many disk-like structures of 30-nm diameter are clearly visible, which are packaged DNA with binding protein in the nucleocapside. (F) Cross-sectional plots comparing the original and deconvolution images of the virion edge in (C) and (D) (white arrow). The virion edge of (D) is visible at the top right. The resolution of the original image is 8 nm, calculated from the normalized intensity width that decreases from 0.75 to 0.25. The resolution of the deconvolution image is 5 nm. Scale bars: (A), (C) and (D) 100 nm, (B) and (E) 50 nm.

First, we used the new method to observe unstained baculoviruses. The baculovirus is rod-shaped, 200–350 nm in length and 60–100 nm in diameter [19]. The genome of baculovirus is packaged in a cigar-shaped nucleocapside in the body [19], [20]. At 80,000× magnification, we observed baculovirus mounted onto the underside of a metal-coated SiN film irradiated with a 4.0-kV acceleration EB. The original image shows black contrast at the virus body (Figure S1). Figure 2A shows the contrast inversion image after application of a two-dimensional (2D) Gaussian filter. Two baculoviruses are visible at the top left and the baculovirus envelope is clearly visible at the bottom right. The nucleocapside is visible at the centre of the virus body (Figure 2B). Another baculovirus scanned under the same conditions shows a similar rod-shaped virus with an envelope (Figure 2C).

These images are slightly blurred because a point-spread function (PSF) under our conditions would be wider than the original EB spot diameter. Therefore, we applied the Lucy–Richardson deconvolution algorithm to the images [21], [22]. The deconvolution image clearly shows a virus and the inner structure of its envelope (Figure 2D).

To investigate the structure of a single stable viral particle, i.e. a virion, we created an expanded pseudo-colour map of a deconvolution image (Figure 2E). This image clearly shows the presence of many disk-like structures, approximately 30 nm in diameter, in a virion, which suggests the presence of packaged DNA with binding protein in the nucleocapside [19], [20]. We estimated the spatial resolutions of the images at the sharp edge of the virion by Reimer’s criteria (Figure 2F). The resolution of the original image was calculated to be 8 nm from the normalized intensity width that decreases from 0.75 to 0.25 (Figure 2F, black line). The resolution of the deconvolution image, 5 nm, is far superior (Figure 2F, red line).

### Observation of Unstained IgM Antibody

**Figure 3:**
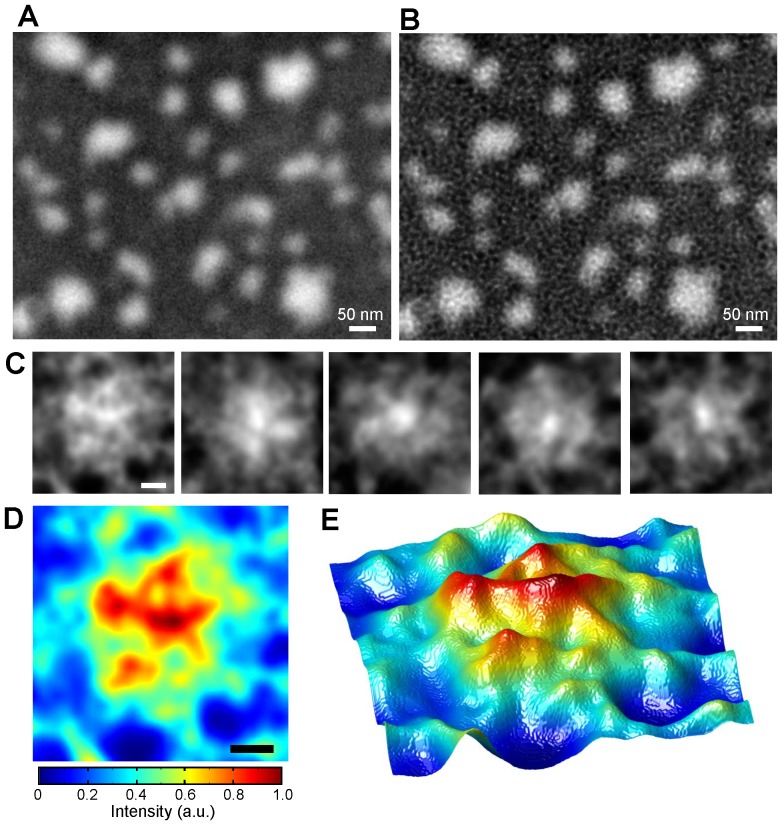
Observation of an unstained IgM antibody. (A) Image of unstained mouse IgM obtained by our method using FE-SEM. The image conditions are as follows: magnification 80,000×, accelerating voltage 3.6 kV EB, application of a 2D Gaussian filter (size 9×9 pixels, σ = 1.5). Many particles of <50-nm diameter are dispersed throughout the area. (B) Super-resolution image of (A) calculated using the Lucy–Richardson deconvolution algorithm. (C) Five individual IgM molecules from the deconvolution images. The molecules are star-shaped pentamers with five IgG arms. (D) Pseudo-colour map of an IgM molecule at the left side of (C). (E) 3D colour map of the same IgM molecule. Note that the five IgG arms are more clearly defined than in the original image. Scale bars: (A) and (B) 50 nm, (C) and (D) 20 nm.

Next, we used the new method to observe unstained IgM antibody molecules. The IgM antibody has a molecular weight of 900 kDa and consists of five IgG antibodies [12], [23]. The IgM pentamer is star-shaped of diameter approximately 45 nm [12], [23]. At 80,000× magnification, we observed IgM mounted onto the underside of a metal-coated SiN film irradiated with a 3.6-kV acceleration EB. Figures 3A and 3B show the original and deconvolution images. Several small particles <50 nm in diameter are dispersed throughout the area. Individual well-separated IgM molecules are visible as star-shaped pentamers with five IgG arms (Figure 3C), similar to the image of unstained IgM molecules obtained by cryo atomic force microscopy (cryo-AFM) [23]. These observations show slightly heterogeneous images, because IgG arms are very flexible [12], [23]. An expanded pseudo-colour map and a 3D colour map (Figures 3D and 3E) clearly show the star-shaped molecules. The centre area shows a high-density circle 20 nm in diameter. This result is consistent with a previously reported cryo-AFM observation [23].

### Observation of Unstained 26S Proteasome

**Figure 4:**
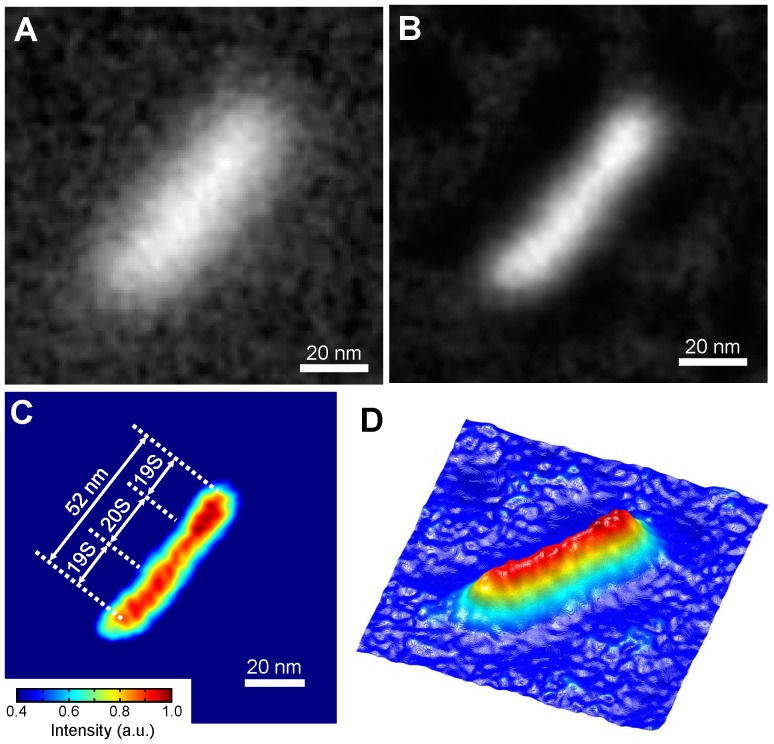
Observation of an unstained 26S proteasome. (A) Image of an unstained human erythrocyte 26S proteasome obtained by our method using FE-SEM. The image conditions are as follows: accelerating voltage 3.6-kV EB, application of a 2D Gaussian filter (size 9×9 pixels, σ = 2). A blurred dumbbell-shaped structure is visible. (B) Deconvolution proteasome image calculated using the Lucy–Richardson deconvolution algorithm. The dumbbell-shaped structure is more clearly defined. (C) Pseudo-colour map of a proteasome molecule of (B). (D) 3D colour map of the same 26S proteasome molecule. Two 19S cups and 20S core are defined. All scale bars: 20 nm.

Finally, we used the new method to observe 26S proteasome molecules. The 26S proteasome contains a barrel-shaped 20S core capped on both ends by 19S particles. The overall structure is dumbbell-shaped of length 45–50 nm and a molecular mass of approximately 2.0 MDa [24]–[27]. At 120,000× magnification, we observed 26S proteasome molecules mounted onto the underside of a metal-coated SiN film irradiated with a 3.6-kV acceleration EB. The original image shows clear contrast and a recognizable dumbbell shape of length 52 nm (Figure 4A and Figure S2). The deconvolution image shows high contrast and a sharper structure (Figure 4B and Figure S2B). A pseudo-colour map and a 3D colour map (Figures 4C and 4D) show the 20S proteasome structure of length 52 nm with two visible 19S ends, which is essentially consistent with other reports [27], [28].

## Discussion

The contrast of unstained biological samples observed by electron microscopy is very low because of weak interaction of the EB with the lightweight atoms. Furthermore, unstained biological specimens suffer serious radiation damage from the EB [7], [8], [15]. Therefore, it is difficult to observe unstained proteins and viruses at high contrast without incurring radiation damage.

Here, we developed a high-contrast low-damage method for observing unstained proteins and viruses with SEM. Unstained specimens are deposited onto the bottom (metal) side of a metal-coated SiN film (Figure 1). High-contrast images of the specimens are obtained at a low EB acceleration of just 3.6–4 kV (Figures 2, 3, and 4). Under these conditions, the irradiated electrons are 80–90% absorbed by the metal-coated film (Figure S3), as calculated by MC simulations [29]. Therefore, this new method protects specimens from electron radiation damage.

The observation images are similar to those obtained by transmission electron microscopy (TEM), depending on the sample volume in the inner structure rather than the surface (Figure 2). Our hypothesis regarding the observation mechanism is shown in Figure 1D. The EB irradiates the top of the SiN film, resulting in a high electric potential gradient in the film probably owing to electrostatic charge at the irradiated position [7]. The gradient forces the SEs generated in the film by the EB to move toward the bottom of the film. Then some of the SEs presumably pass into the sample via quantum tunnelling, enabling observation of the inner structure of the unstained sample without radiation damage.

How can we estimate the electrostatic charge of the EB-irradiated position on the SiN film? Our SEM observation conditions are as follows: EB current 31.3 pA, image size 1,280×1,024 pixels, scanning time 40 s. The number of irradiated electrons per pixel is 5,963. Field emission reportedly occurs in high electric fields of >10 MeV/cm [30]. Thus, the electric potential in the film must reach 10 MeV/cm so that the SEs in the film pass through to the sample via quantum tunnelling. To reach this value in a 50-nm SiN film, the electrification charge potential on the film must be 400 eV. How many electrons are required to reach 400 eV on the film? We assume that the irradiated EB position of the film is equivalent to that of a small capacitor 3 nm in diameter and 50 nm in length. For an ideal capacitor, capacitance *C* = 9.39×10^−21^ F, as calculated from the traditional equation *C* = *ε_0_ε*·*A*/*d*, where SiN relative permittivity *ε* = 7.5 [18], capacitance area *A* = 7.07 nm^2^ and capacitance length *d* = 50 nm. If one electron charges the small capacitor, we obtain charge potential *V* = *q*/*C* ≃ 17 eV. To reach 400 eV on the film, approximately 30 electrons are required to charge the irradiated EB position. Here 5,963 electrons irradiate one pixel. Therefore, the number of electrons required to charge the irradiated position represent only 0.5% of the total number of electrons irradiating a pixel. Its negative charge generates the high-electrical potential gradient in the metal-coated film. This gradient pushed SEs generated under the Au-layer toward the samples, which is contributed to the high-contrast and high-resolution observations. We consider that our hypothesis will be realized on the films.

Our new method achieves a spatial resolution of 8 nm, which is far superior to that achieved by our previous ISEC method [17]. MC simulations suggest that the scattered electron width under the metal-coated SiN film is approximately 40 nm (Figure S4); thus, assuming a normal SE signal on the bottom side of the film, the predicted resolution is 40 nm. However, the measured resolution, 8 nm, is five times better than the predicted value. Therefore, we conclude that the observation mechanism of the new method differs from that of the original ISEC method.

Regarding our hypothesis of SE transmission to the sample by quantum tunnelling, SEs have very low energy because transmission electrons (TEs) are generated from the SEs in the film. Therefore, TE energy is similar to an SE energy of approximately 10 eV. To determine TE energy, we measured the electrical current under the sample with bias potential to the measurement holder (Figure S3). The detection current under the sample decreases when a negative potential is applied to the measurement position and is completely suppressed by a cup potential of −10 eV, indicating that TE energy is <10 eV. These results suggest that TE energy is similar to SE energy.

A spatial resolution of 8 nm is insufficient to observe the details of a protein structure. To improve spatial resolution, we applied the Lucy–Richardson deconvolution algorithm [21], [22] to the obtained images. Doing so improved spatial resolution to 5 nm (Figure 2F). The deconvolution images of unstained IgM and 26S proteasome (Figures 3 and 4) show structural details that are consistent with previous reports [23], [27], [28]. However, for analysis of protein function, spatial resolution must be <2 nm. To approach this level, we are currently constructing a system based on an ultrahigh-resolution SEM with an EB diameter of 1 nm and a new deconvolution algorithm. However, there are still problems associated with the denatured biological samples in the vacuum. One of approaches to this problem, we plan to develop an easy-to-use holder that enables undamaging observation of unstained proteins in the atmosphere and/or water.

In conclusion, unstained proteins and viruses mounted on the underside of metal-coated SiN film give high-contrast images at 8-nm resolution, as observed by an SEM at a low acceleration voltage of 3.6–4.0-kV EB. Application of the Lucy–Richardson deconvolution algorithm improves spatial resolution from 8 to 5 nm. Images of unstained IgM and 26S proteasome proteins show high contrast and clear structural details. Electron radiation damage to unstained biological samples is very low because most irradiated electrons are absorbed into the metal-coated film. Therefore, our novel method can be easily used to observe proteins, viruses and other organic specimens. Our method will be widely contributed to SEM users especially for analysis of the biological samples.

## Materials and Methods

### Metal Coating on a SiN Film

A SiN film (thickness 50 nm) supported by a Si frame window (window size 0.5×0.5 mm square, Si thickness 0.2 mm) (Silson Ltd., UK) was coated with Ni and Au layers by magnetron sputtering (MSP-30T, Vacuum Devices Inc., Japan). Sputtering conditions were as follows–for the Ni layer: thickness 15 nm, Ar pressure 1.1 Pa, sputter current 200 mA, sputter time 15 s; for the Au layer: thickness 10 nm, Ar pressure 1.1 Pa, sputter current 100 mA, sputter time 5 s. The distance between the sputter target and SiN film was 50 mm.

### Sample Preparations

Baculovirus of *Spodoptera litura* NPV was kindly provided by Nippon Kayaku Co. Ltd. (Japan). *Spodoptera litura* NPV powder (10 mg) was dissolved in 1 ml of 10 mM sodium carbonate solution (pH 11.0). After 10 min, a 3-µl portion of baculovirus solution was dropped onto the metal-coated SiN film. After 1 min, the solution on the film was removed with filter paper, and the film was dried at room temperature (23°C) for 5 min.

Mouse IgM antibody solution (code number M079-3) was obtained from Medical & Biological Laboratories Co., Ltd. (Japan). A 2-µl portion of the IgM solution was diluted to 30 µl with distilled water. A 3-µl portion of the diluted solution was dropped onto the metal-coated SiN film. After 2 min, the solution on the film was removed with filter paper, and the film was dried at room temperature (23°C) for 5 min.

A purified 26S proteasome solution from human erythrocyte (code number BML-PW8950) was obtained from Enzo Life Sciences, Inc. (USA). A 2-µl portion of the 26S solution was diluted to 10 µl with distilled water. A 4-µl portion of the diluted solution was dropped onto the metal-coated SiN film. After 2 min, the solution on the film was removed with filter paper, and the film was dried at room temperature (23°C) for 5 min.

### Scanning Electron Microscopy and Image Processing

The stage containing the metal-coated SiN film and sample was transferred to the chamber of a FE-SEM (JSM-7000F, JEOL, Japan). Protein and virus images were captured by the original SE detector of the SEM under the following conditions: magnification 80,000–120,000×, image size 1,280×1,028 pixels, observation time 40 s, working distance 3–4 mm, EB acceleration voltage 3.6–4 kV, current 31.3 pA.

The images were treated with a 2D Gaussian filter (size 11×11 pixels, σ = 1; Matlab R2007b, Math Works Inc., USA). Deconvolution images were calculated from the Lucy–Richardson deconvolution algorithm [21], [22] and the Matlab deconvlucy() function. The parameters of the deconvolution algorithm were filtered by 12 iterations with PSFs as follows: For the baculovirus images, the PSF of 241 square pixels was set to the sum of the 2D Gaussian functions of 60σ and 12σ, where the value of 60σ is half that of 14σ. For the protein images of the IgM and 26S proteasome, the PSFs were set to the Gaussian functions of 60σ and 14σ as well as 100σ and 40σ, respectively. Calculations were performed on a personal computer (Intel Core2 Duo E6850, 3.0 GHz, Microsoft Windows XP).

### Monte Carlo Simulations

Electron trajectories in the metal-coated SiN film were calculated by MC simulations using CASINO version 2.43 software [29]. Material parameters were as follows–for the SiN film: density 3.12 g/cm^3^, thickness 60 nm; for the Ni layer: density 8.9 g/cm^3^, thickness 15 nm; for the Au layer: density 19.3 g/cm^3^, thickness 10 nm. The physical model for simulation was the same as that for our previous study [17]. MC simulation parameters were as follows: 1,000,000 electrons, EB accelerating voltage 3–5 kV, EB spot diameter 3 nm. Simulations were performed on a personal computer (Intel Core2 Duo E6850, 3.0 GHz, Microsoft Windows XP).

## Supporting Information

Figure S1 **Original image of unstained baculovirus obtained by our method using an SEM.** The image was taken at 80,000× magnification and a 4-kV EB accelerating voltage, then filtered by a 2D Gaussian filter (size 9×9 pixels, σ = 1) without contrast reverse. The image shows very clear black contrast. Scale bar: 100 nm.
(TIFF)

Figure S2 **Three individual images of the unstained 26S proteasome of a human erythrocyte obtained by our method.** (A) The images were taken at 120,000× magnification and a 3.6-kV EB accelerating voltage. Scale bar: 20 nm. (B) Three deconvolution images calculated from (A) using the Lucy–Richardson deconvolution algorithm. The images show very clear structure. (C) Pseudo-colour maps of the proteasome molecules of (B).
(TIFF)

Figure S3 **Thermionic SEM measurement of transmission electron current under the film.** (A) Scheme for measuring transmission electron (TE) current by a thermionic SEM (JSM-6390, JEOL, Japan). A Ni–Au-coated SiN film is irradiated by a 4- or 10-kV EB, and TE current is measured at an aluminium hole under the sample using a remote source metre (Keithley 6430, Keithley Inc., USA). The film consists of three components: 50-nm SiN film coated with a 15-nm Ni layer and a 10-nm Au layer. The sample holder is connected to an electric ground. (B) Measured TE current under various bias voltages of the measurement cup. If the energy of TE is less than the bias voltage at the measurement position, the TE does not reach the position owing to electric repulsion force. Therefore, the measured TE current arises only from electrons with energies higher than the bias voltage. For a 10-kV EB, the TE current for a 2-V bias is 165 pA, which falls exponentially to 90 pA as bias decreases to −10 V (red line). For a 4-kV EB, the TE current for a 2-V bias is 48 pA, which falls to 0 pA as bias decreases (black line). (C) Normalized TE current for the 2-V bias of (B). For a 4-kV EB, the TE current for a bias of −10 V is 0, which suggests that TE energy is <10 eV.
(TIFF)

Figure S4 **MC simulations of electron trajectory in the metal-coated SiN film.** (A) Electron energy map of a 3.6-kV EB in the Ni–Au-coated SiN film calculated by MC simulation using CASINO version 2.42 software. The EB diameter is 3 nm. The position where the EB irradiates the film exhibits very high electron energy. Most irradiated electrons scatter and absorb into the film’s metal layer. (B) and (C) Electron energy maps of 4.0- and 4.6-kV EBs, respectively. (D)–(F) Electron energy maps of 3.6-, 4.0- and 4.6-kV EBs, respectively, calculated from the scattered electrons in the film’s bottom Au layer. (G)–(I) Line plots of the scattered widths in the Au layers for (D)–(F). The half-intensity width is 41.8 nm. (J) Line plot of the half-intensity width of the scattered area in the Au layer at 3.0–5.0-kV EB. The half-intensity width is approximately 40 nm. (K) TE rates in the metal-coated SiN film for EB voltages of 3.0–5.0 kV, calculated by MC simulation. TE rate increases linearly with EB voltage. At EB voltages of 3.6 and 4.0 kV, irradiated electrons transmit through the film at rates of 10% and 20%, respectively.
(TIFF)
