# Supplementary figures and images for: High-Contrast Observation of Unstained Proteins and Viruses by Scanning Electron Microscopy
Source: PLoS One. 2012 Oct 8;7(10):e46904. doi: 10.1371/journal.pone.0046904 (PMC3466209; doi:10.1371/journal.pone.0046904)

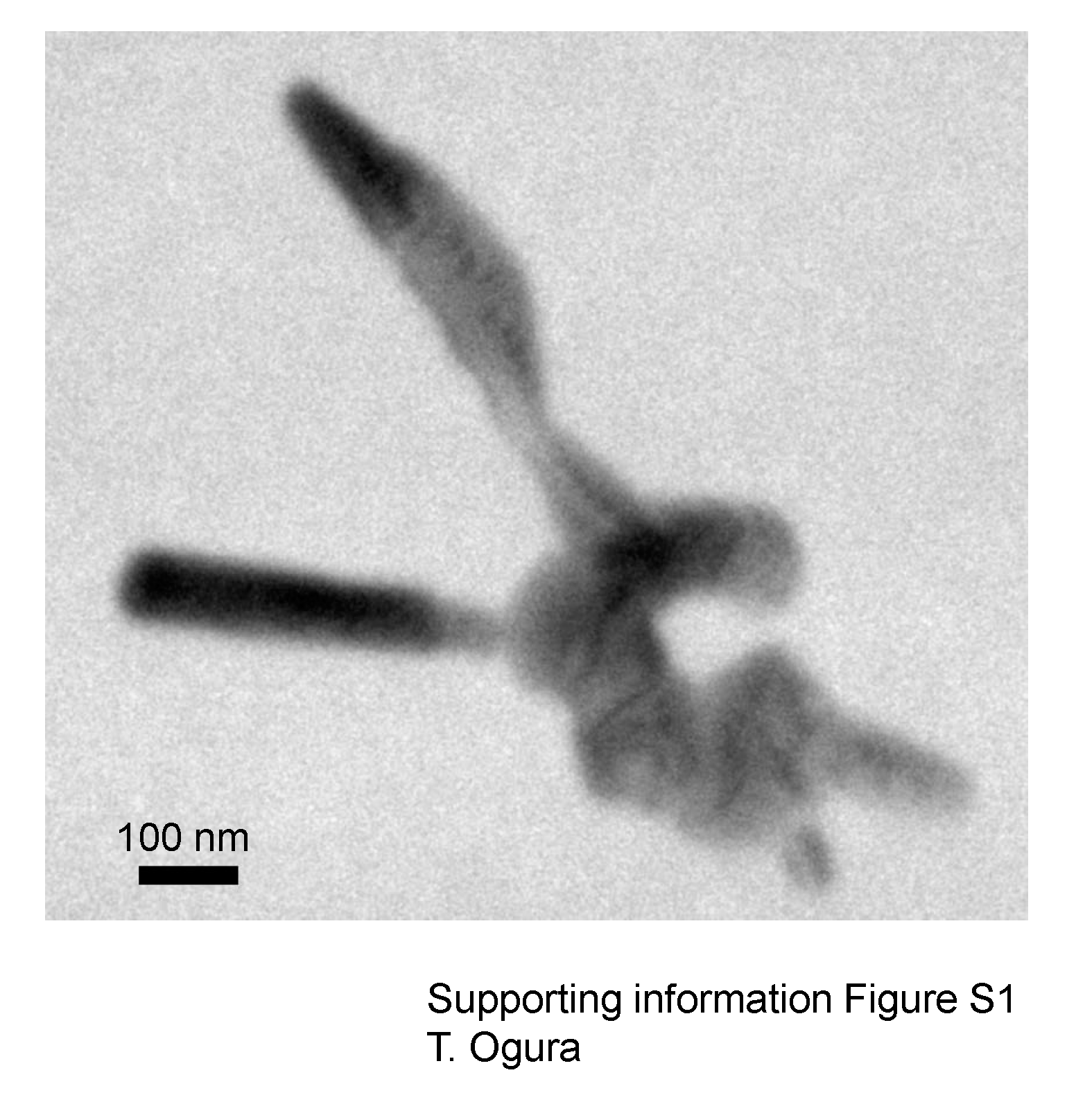

Supplement: Figure S1 — Original image of unstained baculovirus obtained by our method using an SEM. The image was taken at 80,000× magnification and a 4-kV EB accelerating voltage, then filtered by a 2D Gaussian filter (size 9×9 pixels, σ = 1) without contrast reverse. The image shows very clear black contrast. Scale bar: 100 nm. (TIFF) [file pone.0046904.s001.tif]

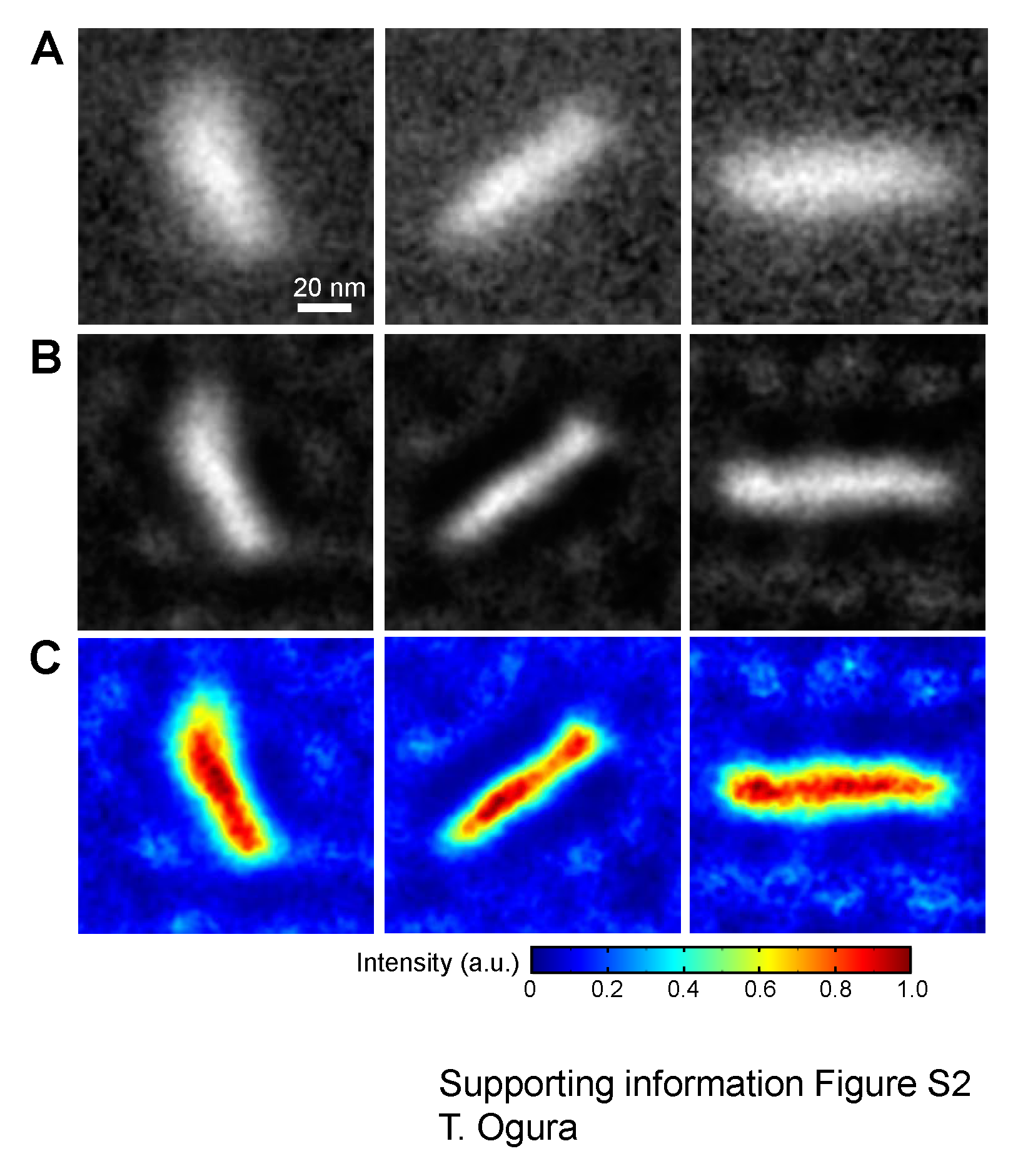

Supplement: Figure S2 — Three individual images of the unstained 26S proteasome of a human erythrocyte obtained by our method. (A) The images were taken at 120,000× magnification and a 3.6-kV EB accelerating voltage. Scale bar: 20 nm. (B) Three deconvolution images calculated from (A) using the Lucy–Richardson deconvolution algorithm. The images show very clear structure. (C) Pseudo-colour maps of the proteasome molecules of (B). (TIFF) [file pone.0046904.s002.tif]

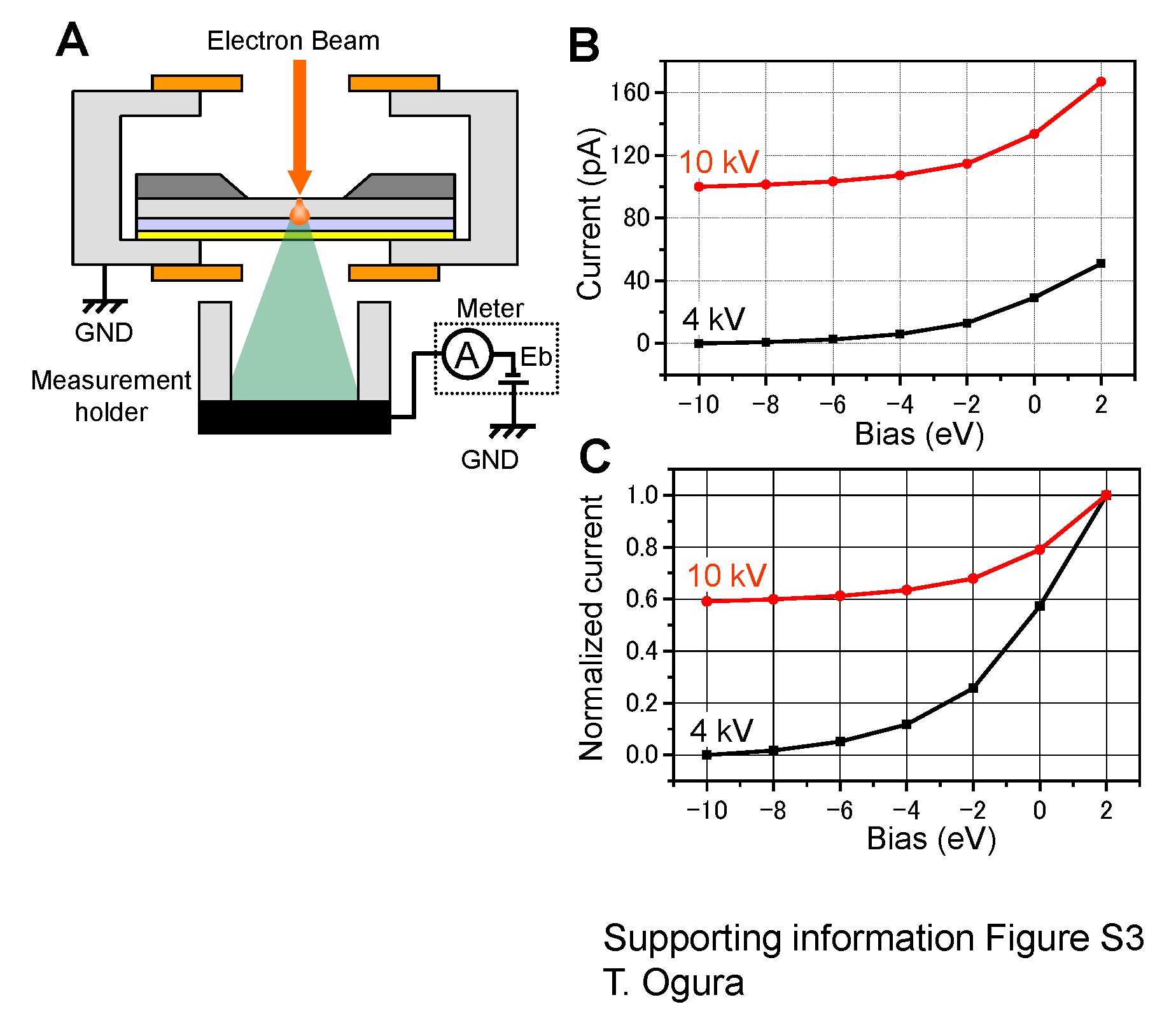

Supplement: Figure S3 — Thermionic SEM measurement of transmission electron current under the film. (A) Scheme for measuring transmission electron (TE) current by a thermionic SEM (JSM-6390, JEOL, Japan). A Ni–Au-coated SiN film is irradiated by a 4- or 10-kV EB, and TE current is measured at an aluminium hole under the sample using a remote source metre (Keithley 6430, Keithley Inc., USA). The film consists of three components: 50-nm SiN film coated with a 15-nm Ni layer and a 10-nm Au layer. The sample holder is connected to an electric ground. (B) Measured TE current under various bias voltages of the measurement cup. If the energy of TE is less than the bias voltage at the measurement position, the TE does not reach the position owing to electric repulsion force. Therefore, the measured TE current arises only from electrons with energies higher than the bias voltage. For a 10-kV EB, the TE current for a 2-V bias is 165 pA, which falls exponentially to 90 pA as bias decreases to −10 V (red line). For a 4-kV EB, the TE current for a 2-V bias is 48 pA, which falls to 0 pA as bias decreases (black line). (C) Normalized TE current for the 2-V bias of (B). For a 4-kV EB, the TE current for a bias of −10 V is 0, which suggests that TE energy is <10 eV. (TIFF) [file pone.0046904.s003.tif]

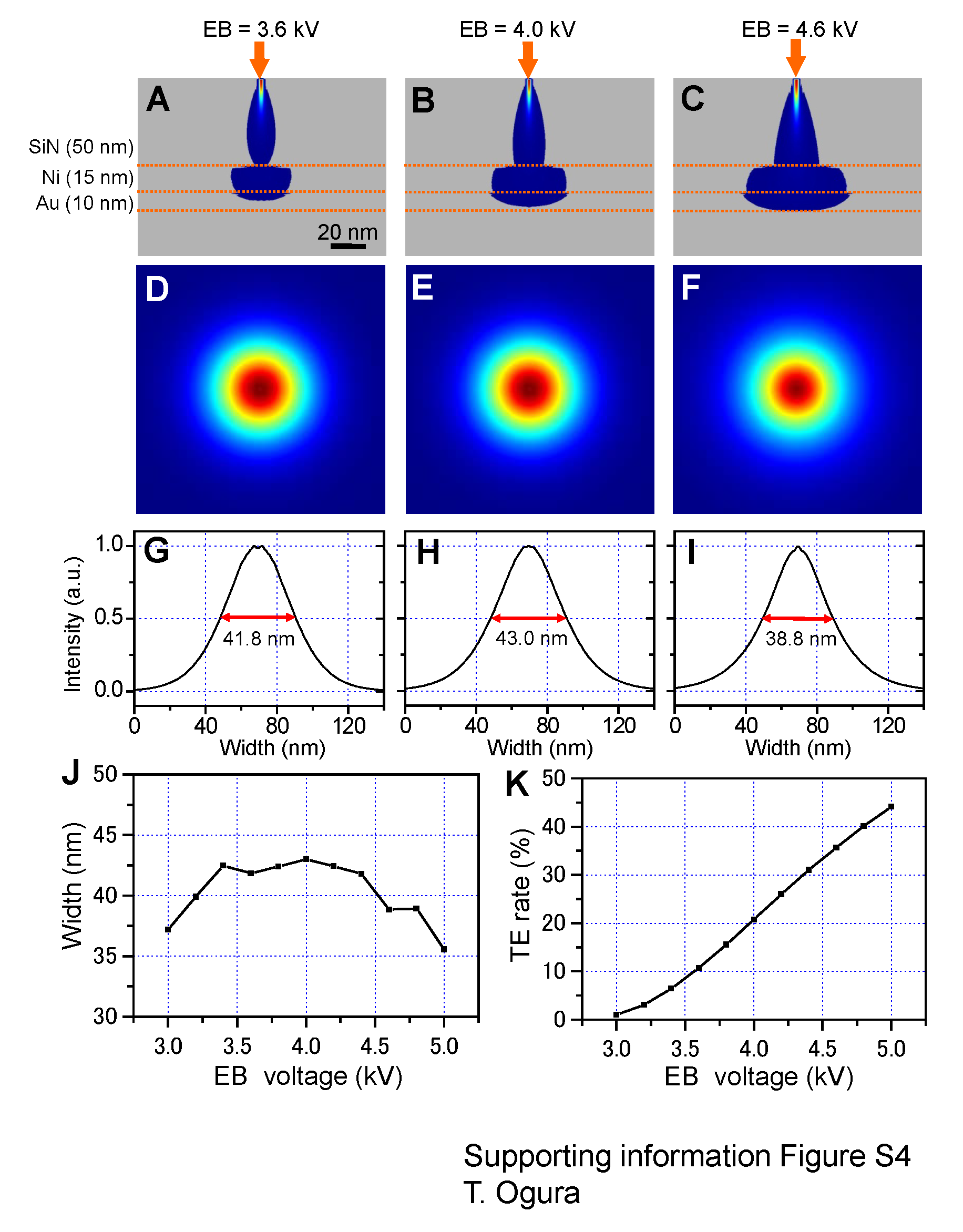

Supplement: Figure S4 — MC simulations of electron trajectory in the metal-coated SiN film. (A) Electron energy map of a 3.6-kV EB in the Ni–Au-coated SiN film calculated by MC simulation using CASINO version 2.42 software. The EB diameter is 3 nm. The position where the EB irradiates the film exhibits very high electron energy. Most irradiated electrons scatter and absorb into the film’s metal layer. (B) and (C) Electron energy maps of 4.0- and 4.6-kV EBs, respectively. (D)–(F) Electron energy maps of 3.6-, 4.0- and 4.6-kV EBs, respectively, calculated from the scattered electrons in the film’s bottom Au layer. (G)–(I) Line plots of the scattered widths in the Au layers for (D)–(F). The half-intensity width is 41.8 nm. (J) Line plot of the half-intensity width of the scattered area in the Au layer at 3.0–5.0-kV EB. The half-intensity width is approximately 40 nm. (K) TE rates in the metal-coated SiN film for EB voltages of 3.0–5.0 kV, calculated by MC simulation. TE rate increases linearly with EB voltage. At EB voltages of 3.6 and 4.0 kV, irradiated electrons transmit through the film at rates of 10% and 20%, respectively. (TIFF) [file pone.0046904.s004.tif]
